# Large Scale *In Vivo* Recording of Sensory Neuron Activity with GCaMP6

**DOI:** 10.1523/ENEURO.0417-17.2018

**Published:** 2018-04-06

**Authors:** Kim I. Chisholm, Nikita Khovanov, Douglas M. Lopes, Federica La Russa, Stephen B. McMahon

**Affiliations:** Neurorestoration Group, Wolfson Centre for Age-Related Diseases, King’s College London, London SE1 1UL, United Kingdom

**Keywords:** dorsal root ganglia, genetically encoded calcium indicators, *in vivo* imaging, nociception, pain, primary afferents

## Abstract

Greater emphasis on the study of intact cellular networks in their physiological environment has led to rapid advances in intravital imaging of the central nervous system (CNS), while the peripheral system remains largely unexplored. To assess large networks of sensory neurons, we selectively label primary afferents with GCaMP6s in male and female C57bl/6 mice and visualize their functional responses to peripheral stimulation *in vivo*. We show that we are able to monitor the activity of hundreds of sensory neurons simultaneously, with sufficient sensitivity to detect, in most cases, single action potentials with a typical rise time of around 200 ms, and an exponential decay with a time constant of approximately 700 ms. With this technique we are able to characterize the responses of large populations of sensory neurons to innocuous and noxious mechanical and thermal stimuli under normal and inflammatory conditions. We demonstrate that the majority of primary afferents are polymodal with between 50–80% of thermally sensitive DRG neurons responding also to noxious mechanical stimulation. We also specifically assess the small population of peripheral cold neurons and demonstrate significant sensitization to cooling after a model of sterile and persistent inflammation, with significantly increased sensitivity already at decreases of 5°C when compared to uninflamed responses. This not only reveals interesting new insights into the (patho)physiology of the peripheral nervous system but also demonstrates the sensitivity of this imaging technique to physiological changes in primary afferents.

## Significance Statement

Most of our functional understanding of the peripheral nervous system has come from single unit recordings. However, the acquisition of such data are labor intensive and usually “low yield.” Moreover, some questions are best addressed by studying populations of neurons. To this end we report on a system that monitors activity in hundreds of single sensory neurons simultaneously, with sufficient sensitivity to detect in most cases single action potentials. We use this technique to characterize response properties to natural and electrical stimuli and to demonstrate polymodality in the majority of neurons as well as their sensitization under inflammatory conditions. We therefore believe this approach will be very useful for the study of the somatosensory system in general, and pain in particular.

## Introduction

Primary somatosensory neurons and their cell bodies located in the dorsal root ganglion (DRG) are functionally, anatomically, physiologically and genetically diverse (Kandel et al., 2013; Usoskin et al., 2015). These neurons are critical for a large number of distinct sensations including, but not limited to, touch, pain, itch, proprioception and temperature. Our knowledge regarding the encoding properties of these neurons is largely derived from single unit recording studies of individual afferent fibers, work that is hampered by the low throughput of such data acquisition.

The advent of genetically encoded calcium indicators has opened up the possibility for large scale optical assessment of the functional and morphologic characteristics of entire neuronal networks with good spatial and temporal sensitivity. These techniques have been applied to a variety of CNS structures, including sensory, motor, and visual cortex and spinal cord (Stosiek et al., 2003; Dombeck et al., 2007; Flusberg et al., 2008; Tian et al., 2009; Johannssen and Helmchen, 2010; Ghosh et al., 2011; Chen et al., 2012, 2013; Zariwala et al., 2012; Sun et al., 2013; Dana et al., 2014; Sekiguchi et al., 2016) and, very recently, some peripheral networks (Barretto et al., 2015; Williams et al., 2016; Wu et al., 2015). In recent months, a few groups have also described the application of *in vivo* imaging to the DRG (Emery et al., 2016; Kim et al., 2016; Smith-Edwards et al., 2016), but due to the novelty of the technique the peripheral nervous system still remains relatively unexplored.

Indeed, it is evident from this small string of papers that the application of *in vivo* imaging to the peripheral nervous system is still an evolving field. For example, an initially slower version of the calcium indicator GCaMP, as well as very slow image acquisition speeds, may have hampered a more detailed analysis of the pathophysiology of the peripheral nervous system (Kim et al., 2016; Smith-Edwards et al., 2016), while small numbers of sampled neurons could reduce the benefits inherent to this approach (Emery et al., 2016; Smith-Edwards et al., 2016). For example, one such recent publication suggested that the vast majority of primary afferents exhibit modality specificity (Emery et al., 2016). This is in stark contrast to the frequently observed (and widely reported) nociceptive polymodality seen using microneurography and electrophysiology, in addition to multiple lines of *in vitro* evidence (as reviewed in chapter 1 of McMahon et al., 2013). Here, we re-examine this claim using large scale *in vivo* GCaMP imaging, and confirm traditional views of widespread primary afferent polymodality.

We present an enhanced method which provides for the first time the added benefits of being able to sample from hundreds of neurons simultaneously, at acquisition frequencies above 1 Hz, using very low light levels with no detectable photo-bleaching and an increased resistance to sample movement. These features make this technique useful to the study of large populations of heterogeneous neurons responding variably across time. With this technique, we are therefore able to sample the diverse array of DRG neurons in response to acute and persistent pain states *in vivo*.

## Materials and Methods

For all experiments, aside from polymodality experiments, we used adult C57BL/6J mice (Envigo) and expressed GCaMP6s in sensory neurons via intrathecal AAV9. For comparison, we also studied responses in Cre-dependent C57BL/6 GCaMP6s mice (The Jackson Laboratory, stock no: 028866) crossed with Advillin-CreERT2 (courtesy of John Wood), and also in Snap25-2A-GCaMP6s-D (The Jackson Laboratory, stock no: 025111) in experiments of polymodality only (Fig. 4*C*). The animals weighed 20–35 g at the time of experimentation. Both male and female mice were housed on a 12/12 h light/dark cycle with a maximum of 8 mice per cage, with food and water available *ad libitum*. All experiments were performed in accordance with the United Kingdom Home Office Animals (Scientific Procedures) Act (1986).

### Intrathecal administration of AAV9-GCaMP6s

C57BL/6J mice were anesthetized with isoflurane (∼2% in oxygen), and Carprieve (0.025 mg; Norbrook Laboratories) was administered subcutaneously for postoperative pain management. Mice were maintained at around 37°C using a homeothermic heating mat. An incision was made in the skin over the lumbar region and muscle was removed to expose the intervertebral membrane between T12 and T13 vertebrae. The region surrounding the lumbar enlargement provides easier access to the intervertebral area and ensures minimal damage. A small cut was made in the membrane and the underlying dura to insert a small catheter of 0.2-mm diameter (Braintree Scientific) in the caudal direction, through which 5 µl of AAV9.CAG.GCaMP6s.WPRE.SV40 (UPENN Vector Core, AV-1-PV2833, 1.1 × 1013 gc/ml) was infused into the intrathecal space at 1.2 µl/min. Due to the length of the inserted cannula, the infusion was close to the L4 DRGs. The catheter was left in place for 2 min before slow withdrawal. The incision was closed and mice were allowed to recover for between 14–37 d.

### Tamoxifen dosing

Tamoxifen (T5648; Sigma-Aldrich) was dissolved in 100% ethanol to a concentration of 97.5 µg/µl and further dissolved to a working concentration of 7.8 µg/µl in wheat germ oil (Denk et al., 2015). This was placed on a shaker at room temperature for 2–3 hr and subsequently stored at −20°C. To induce GCaMP expression, mice carrying the Advillin-CreERT2 allele received 75 mg/kg tamoxifen intraperitoneally once daily for 3 d. We waited for a minimum of 14 d to allow induction and accumulation of GCaMP6.

### *In vivo* imaging of sensory neuron activity using GCaMP responses

For *in vivo* imaging, mice were anesthetized using urethane (12.5% w/v). An initial dose of 37.5 mg (in a volume of 0.3 ml) was given IP. Further doses were given at ∼15- to 20-min intervals, depending on hindlimb and corneal reflex activity, until surgical depth was achieved. The core body temperature was maintained close to 37°C using a homeothermic heating mat with a rectal probe (FHC). A tracheal catheter was installed and the mice breathed spontaneously. Animals were hydrated with 0.5-ml sterile normal saline (0.9%) administered subcutaneously. An incision was made in the skin on the back and the muscle overlying the L3, L4, and L5 DRG was removed. The bone around the L4 DRG was carefully removed in a caudal-rostral direction and the underlying epineurium and dura mater over the DRG were washed and moistened with normal saline. The position of the mouse was varied between prone and lateral recumbent to orient the DRG in a more horizontal plane. The exposure was then stabilized at the neighboring vertebrae using spinal clamps (Precision Systems and Instrumentation) attached to a custom-made imaging stage. The exposed cord and DRG were covered with silicone elastomer (World Precision Instruments, Ltd) to avoid drying and to maintain a physiologic environment. The mouse was then placed under the Eclipse Ni-E FN upright confocal/multiphoton microscope (Nikon) and the microscope stage was variably diagonally orientated to optimize focus on the DRG. The ambient temperature during imaging was kept at 32°C throughout. All images were acquired using a 10× dry objective. To obtain confocal images a 488-nm Argon ion laser line was used, while a Coherent Chameleon II laser was tuned to 920 nm for multiphoton imaging. GCaMP signal was collected at 500–550 nm. Time series recordings were taken with an in-plane resolution of 512 × 512 pixels and a fully open pinhole for confocal image acquisition. Image acquisition varied between 1 and 16 Hz depending on the experimental requirements and signal strength.

### Activation of sensory neurons with electrical stimuli

In some animals, the sciatic nerve on the side ipsilateral to the DRG being imaged was exposed through blunt dissection. A custom-made cuff electrode with Teflon insulated silver wire (Ø 0.125 mm; Advent Research Materials) was placed underneath and around the sciatic nerve. The preparation was isolated and stabilized using dental silicon impression compound (Heraeus Kulzer). A biphasic stimulator (World Precision Instruments) was used to deliver either individual or trains of square wave current pulses to the sciatic nerve. Pulses of 250-µs duration and 250-µA amplitude were used to activate myelinated (A) fibers in the sciatic nerve, and supramaximal pulses of 1-ms duration and 5-mA amplitude were used to activate all afferent fibers, both myelinated (A) and unmyelinated (C) axons.

### Activation of sensory neurons with thermal stimuli

A Peltier device (TSAII, Medoc) with a 16 × 16 mm probe was placed onto the plantar surface of the hind paw ipsilateral to the DRG being imaged. The temperature of the block was increased from a baseline temperature of 32°C to 50°C or decreased from 32°C to 4°C. Temperature changes occurred as either a ramp of 1.5°C/s to the target temperature (maintained for 10 s) before returning to baseline at 4°C/s, or as increasing/decreasing incremental temperature ramps. Consecutive increments occurred as steps of 2°C in the case of an increase, and 5°C during a decrease (with a final drop of 3°C from 7° to 4°C) and a minimum of 90 s between incremental changes. Each individual increment involved a temperature change of 2°C/s, a holding temperature for 5 s and a return to baseline at 4°C/s.

### Activation of sensory neurons with mechanical and chemical stimuli

Mechanical stimulation consisted of brushing or pinching of the plantar surface of the ipsilateral hind paw. To achieve the stimulation of the maximal number of receptive fields the entire plantar surface was pinched with blunt forceps across most of the surface of the paw. An effort was made to stimulate similar areas across different experiments to activate a comparable number of sensory fibers.

In some experiments, sterile normal saline and formalin (1.85% in saline) was injected at a volume of 20 µl into the middle of the plantar surface of the ipsilateral hind paw. Responses of L4 DRG neurons were monitored for at least 30 min after injection of saline followed by an injection of formalin and a further 30 min of observation.

### Behavior assessment after formalin injection

For behavioral experiments, mice were placed in a Perspex chamber on a wire mesh floor and allowed to acclimatize for at least 30 min. After acclimatization, mice were lightly restrained and 20 μl of 1.85% formalin in 0.9% saline solution was injected subcutaneously into the plantar surface of the hind paw using a 31-Gauge syringe. The animal was then put back into the chamber and its behavior was recorded for 30 min. Pain behavior (time spent flinching, jerking or licking the injected paw) was quantified from video footage, using Etholog Software (Ottoni, 2000) in blocks of 5 min. All behavioral experiments were conducted during the light cycle of the day.

### UVB irradiation

Mice were anesthetized with ketamine (75 mg/kg in saline, Narketan, Vetoquinol) and medetomidine (0.5 mg/kg in saline, Dormitor, Vetoquinol) and placed under a black cloth for protection. Their eyes were moistened and protected with eye gel (Viscotears, Liquid Gel, Novartis) and the plantar surface of the left paw was exposed through slits in the material and immobilised using tape. The paws of mice were exposed to 3000 mJ/cm^2^ over ∼40 min [the duration of exposure depended on the strength of the UVB lamp -TL01 fluorescent bulbs (maximum wavelength, 311 nm), assessed before each irradiation using a photometer]. Control animals were anesthetized but not irradiated. Animals were allowed to recover for 48 h before *in vivo* imaging.

### Evaluation of transfection efficiency of DRGs

Fourteen days after intrathecal injection of AAV9-GCaMP6s, four mice were terminally anesthetized and transcardially perfused with PBS followed by 4% paraformaldehyde (PFA). The L4 DRGs were removed and postfixed for 2 h in 4% PFA before cryoprotection in 30% sucrose (with 0.02% sodium azide) for 24 h. They were then embedded in Optimal Cutting Temperature (Tissue-Tek), cut into 10-µm sections at -20°C and mounted onto glass slides.

Once dried, DRG slices were rehydrated and blocked with 10% serum for 1 h before incubation with primary antibodies against β-III-tubulin (primary afferent marker; 1:1000, Promega, G712A), GFP (to visualize GCaMP6s; 1:1000, Abcam, ab13970), neurofilament 200 (NF200; large myelinated neurons; 1:160, Sigma Aldrich, N4142), calcitonin gene-related peptide (CGRP; small peptidergic neurons; 1:500, Enzo Life Sciences, CA1134), and isolectin B4 (IB4; conjugated to Alexa Fluor 647; small, non-peptidergic fibers; 1:250, Invitrogen, I32450) overnight at room temperature. Slides were then incubated with the appropriate fluorophore-conjugated secondary antibodies (goat anti-chicken, Alexa Fluor 488, Invitrogen, A-11039, for GFP; goat anti-rabbit, Alexa Fluor 594, Invitrogen, A-11037, for CGRP and NF200; goat anti-mouse, Alexa Fluor 647, Invitrogen, A-32728, for β-III-tubulin, all used at 1:1000 dilution) for 2 h at room temperature. Slides were coverslipped using DAPI-containing media (Fluoromount-G with DAPI, eBioscience) and imaged with an LSM 710 laser-scanning confocal microscope (Zeiss).

### Experimental design and statistical analysis

Drift in time-lapse recordings were corrected using NIS Elements AR 4.30.01 (Nikon, align application). Further image processing was done using Fiji/ImageJ version 1.48v, and graphing and statistical analysis was undertaken with a combination of Microsoft Office Excel 2013, IBM SPSS Statistics 23 package and RStudio 0.99.893. All tests conducted were two-tailed, and *p* < 0.05 was considered significant. Statistical tests and sample sizes are fully recorded in the appropriate figure legends.

To generate traces of calcium signals from time lapse images, regions of interest (ROIs) surrounding cell bodies were chosen using a free hand selection tool in Fiji. ROIs were chosen with minimal overlap to ensure less interference from surrounding somata.

A region of background was selected and its signal subtracted from each ROI. To generate normalised data, a baseline period of fluorescence was recorded for each ROI and changes from this baseline fluorescence were calculated as ΔF/F.

$$\Delta F/F=\frac{F_{t}-F_{0}}{F_{0}}$$

Where F_t_ is the fluorescence at time t and F_0_ is the fluorescence average over a baseline period. Unless otherwise stated ΔF/F is expressed as a percentage.

The threshold for a positive response was taken as an average signal of 70% above baseline fluorescence plus 4 SD.

For tests of polymodality, a high level of stringency was used. To ensure that overlapping cell bodies were not contaminating the signal, and that low levels of response were not missed, all thermally responsive neurons were double checked visually for their response to pinch.

For histologic analysis, to assess the success of labeling with AAV9, six to eight neuronal cell bodies with the lowest fluorescence were selected and their average intensity plus 1 SEM was considered the cutoff for a positive signal against which all other neurons were assessed.

## Results

### Intrathecal injections of AAV9 label a large and representative subset of neurons in L4 DRGs

**Figure 1.**
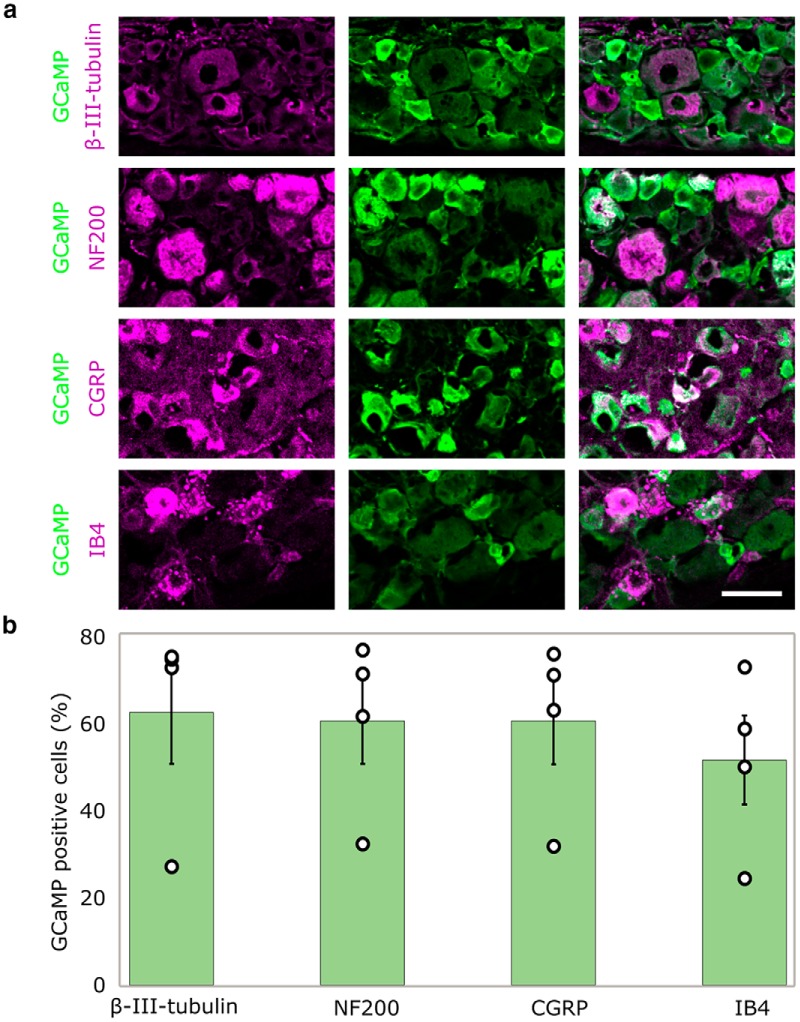
Intrathecal injections label a representative sample of DRG neurons. ***A***, Representative image of GCaMP-positive cells (green) as a proportion of all β-III-tubulin, NF200, CGRP, and IB4 immunoreactive neurons (magenta) after intrathecal injection. Scale bar: 50 µm. β-III-tubulin is a pan neuronal marker; NF200 labels large myelinated neurons; CGRP labels small peptidergic neurons; IB4 labels small, non-peptidergic fibers. ***B***, Quantification of ***A***; the percentage of β-III-tubulin, NF200, CGRP, and IB4 immunoreactive cells that are also positive for GCaMP; *n* = 4 mice. Green bar graphs represent mean ± SEM while individual data points are displayed as empty circles.

To assess efficiency of transfection, four mice, intrathecally injected with AAV9-GCaMP6, were studied histologically 14 d postinjection. In all animals, GCaMP labeling was detected using immunohistochemistry with a GFP antibody, and all neurons were identified with a β-III-tubulin antibody. A mean of 62 ± 12% of β-III-tubulin antibody positive neurons were labeled with GCaMP6s following intrathecal injection (Fig. 1). We also observed that 100% of GCaMP6s labeled cells were β-III-tubulin-positive, which suggests that transduction was limited to neurons. Further, we were able to stain a representative population of large myelinated (NF200-positive, 60 ± 10%), small peptidergic (CGRP-positive, 60 ± 10%), and small non-peptidergic (IB4-positive, 51 ± 10%) neurons with GCaMP, suggesting pan-neuronal tropism (Fig. 1).

### *In vivo* imaging of GCaMP6-labeled neurons: confocal versus two-photon microscopy

**Figure 2.**
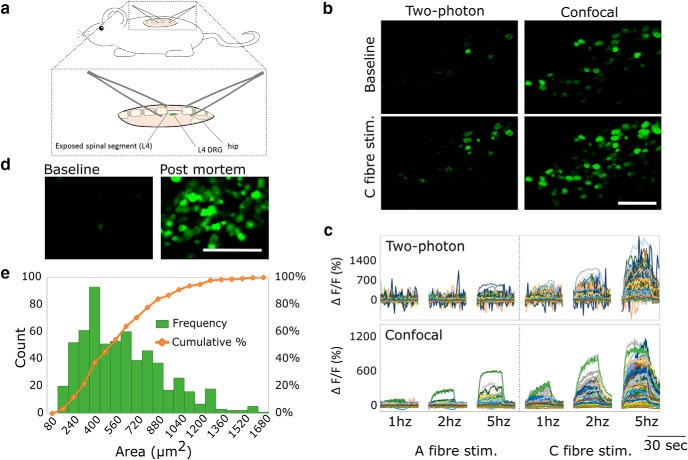
GCaMP labeled DRG neurons can be visualized *in vivo* using standard confocal microscopy. ***A***, Diagram showing the imaging set-up. The L4 DRG is exposed in a deeply anesthetized mouse, and the spinal column on either side of the exposed DRG is stabilized for *in vivo* confocal/two-photon imaging using spinal clamps attached to a custom-made stage. ***B***, Sample images of the DRG *in vivo* acquired under confocal (open pinhole) or two-photon mode. Confocal microscopy with an open pinhole reveals cellular GCaMP signal, which is lost in two-photon acquisition mode. Due to the more restricted slice thickness of multiphoton microscopy (left panel), fewer neurons are visible in the same field of view compared to when confocal microscopy is used with an open pinhole (right panel). This is particularly obvious during stimulation of the sciatic nerve (lower panel). Scale bar: 200 µm. ***C***, Fluorescence traces of ***B***. The sciatic nerve was stimulated at 1, 2, and 5 Hz at both A- and C-fiber strengths to achieve a representative view of different response amplitudes both during confocal and multiphoton acquisition. In this preparation, a cleaner signal is generated when confocal microscopy with an open pinhole is used compared to two-photon microscopy. ***D***, Sample images of the DRG at baseline and following neuronal calcium accumulation >30 min after death. GCaMP6s provides a large dynamic range over which to detect signal changes *in vivo*. Very little signal is evident when the animal is unstimulated (baseline) while a large increase in signal strength is evident following intracellular calcium accumulation >30 min after death (postmortem). Scale bar: 200 µm. ***E***, Frequency histogram and cumulative sum percentage of differentially sized cell bodies shows a skewed distribution with a larger percentage of smaller somata; *n* = 3456 neurons in *n* = 13 mice.

We exposed and visualized the L4 DRG *in vivo* and were able to detect consistent labeling as early as two weeks after AAV9-GCaMP6 injection. The primary stability for imaging was provided by clamping the spinous process above and below the L4 vertebra with forceps (Fig. 2*A*). Rotation of the mouse and spinal cord position in the horizontal axis optimised the orientation of the DRG toward the optical plane of the objective and ensured maximal focus of DRGs during *in vivo* imaging.

We found that confocal microscopy with a maximally opened pinhole offered several advantages over multiphoton microscopy in our preparation. Confocal imaging provided more available signal, including from out-of-focus neurons (Fig. 2*B*). As a result, more information could be extracted from a larger number of somata (Fig. 2*B*,*C*). In addition, the greater optical slice thickness reduced the negative effects of motion and provided more stable traces of fluorescence over time (Fig. 2*C*), which is particularly important when stimulation induces some movement (e.g., with electrical stimulation). Furthermore, the increased signal (compared with the thinner z slices available with two-photon imaging) required lower laser strengths and offered faster acquisition. Using this technique, we were able to image at >16 Hz for over an hour without visible photo damage. These relative benefits of confocal microscopy in this preparation may be especially pronounced due to the absence of a coverslip and the curved shape of the DRG, which result in a less regular air/sample interface and more out of focus tissue.

Using the approach outlined above it was possible to assess our minimal and maximal imaging range through comparison of cellular fluorescence at baseline (without any stimulation) and following cellular calcium accumulation >30 min after death (Fig. 2*D*). This revealed a large dynamic range over which we could visualize changes in intracellular calcium.

Using a 10× objective lens, we were typically able to image over 200 neurons simultaneously per DRG. Estimation of cell size shows an expected unimodal distribution with a majority of small diameter neurons and a tail of fewer, large diameter somas (Fig. 2*E*).

In the absence of stimulation most neurons had stable fluorescence with a SD of around 7%. Only 79 of 1616 cell bodies (∼5%) showed significant spontaneous fluctuations, which are likely to represent ongoing activity (from comparisons with evoked responses).

### Assessing the sensitivity of GCaMP6s sensory neuron imaging with electrical stimulation of the sciatic nerve

Because of the novelty of our technique it was necessary to establish the relationship between action potential firing and calcium transients in DRG neurons. We took advantage of the fact that action potentials can be elicited in DRG neurons with a precisely controlled frequency and temporal pattern via electrical stimulation of peripheral nerves.

We used direct electrical stimulation of the sciatic nerve (which contains axons of the large majority of L4 DRG cell bodies), to induce activity in either large myelinated sensory neurons (A-fibers) or large and small afferents (A- and C-fibers) at different frequencies. These stimulations can induce an action potential that can propagate into the DRG and depolarize the cell body.

**Figure 3.**
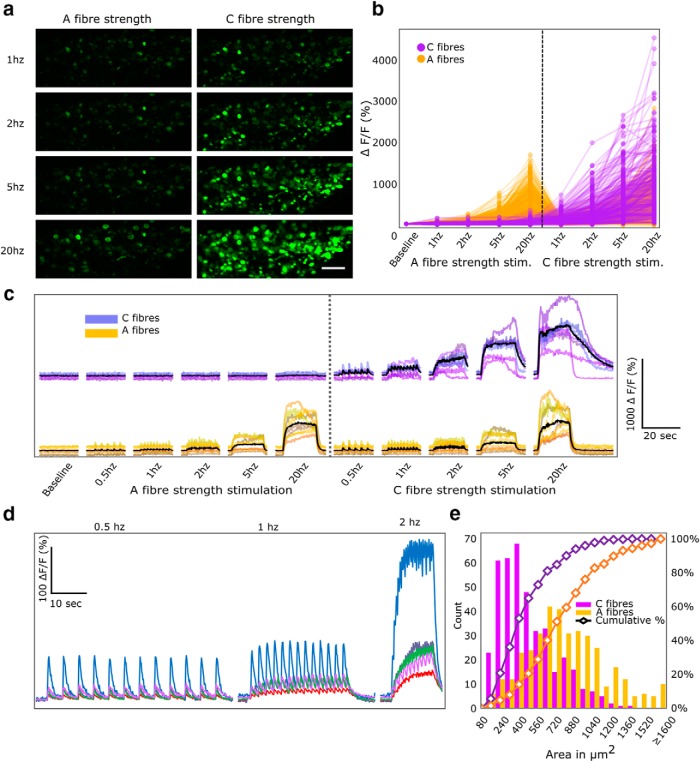
Electrical stimulation of the sciatic nerve leads to an increase in calcium signals in the DRG in an intensity and frequency dependent manner. ***A***, Images of DRG cell bodies during direct stimulation of the sciatic nerve, at different frequencies and intensities. Scale bar: 200 µm. ***B***, Traces of individual cell bodies responding to electrical stimulation. Yellow lines represent neurons that respond to both A- and C-fiber strength stimulation and purple lines represent neurons that respond to C-fiber strength stimuli only. Data points displayed at averaged intensity over the period of stimulation; *n* = 774 neurons in *n* = 6 mice. ***C***, Representative traces of GCaMP fluorescence during electrical stimulation. Electrical stimulation at A- and C-fiber strength resulted in activation of distinct subsets of neurons in a frequency dependent manner. Black lines represent averaged data. ***D***, Representative traces of GCaMP fluorescence during stimulation at C-fiber strength at low frequencies (0.5, 1, and 2 Hz) and high frame acquisition rate (8 Hz). Single action potentials generated by single electrical pulses to the sciatic nerve could be detected in the DRG. Each trace represents fluorescence from a single cell. ***E***, Size distribution of cell bodies that respond to A- and C-fiber strength stimulation (yellow) compared to cell bodies that respond only to C-fiber strength stimuli (purple) and the cumulative sum of their sizes. Neurons responsive to A- and C-fiber strength stimulation are significantly larger compared to neurons which only respond to C-fiber strength stimuli: two-sample Kolmogorov–Smirnov test, *n* = 776 neurons in *n* = 6 mice, *p* < 0.001. See also Video 1 for a recording of neuronal responses to electrical stimulation of the sciatic nerve.

We found that, with A-fiber strength stimulation, a subset of electrically responsive L4 DRG neurons showed increased fluorescence (Fig. 3*A*–*C*). The average magnitude of the A-fiber maximal response at 20 Hz was 654 ± 974% of basal fluorescence. Supramaximal sciatic nerve stimulation activated the same neurons, but additionally recruited the large majority of remaining labeled neurons in the L4 DRG. These additional cell bodies, which only responded to high intensity stimulation, were presumed to be neurons with C-fiber axons. They showed an average magnitude of 868 ± 765% of basal fluorescence at their maximal response during 20-Hz stimulation. In cell bodies of both A- and C-fibers, the fluorescence intensity increased with stimulus frequency (Fig. 3*B*,*C*), suggesting that GCaMP6 fluorescence intensity provides a useful proxy for frequency of action potential firing. See also Video 1 for a recording of neuronal responses to electrical stimulation of the sciatic nerve.

Video 1. Electrical stimulation of the sciatic nerve. L4 DRGs response to electrical stimulation of the ipsilateral sciatic nerve. GCaMP fluorescence was intensity and frequency dependent with recruitment of predominantly large diameter cell bodies during A-fiber strength stimulation and recruitment of additional, smaller diameter cell bodies with C-fiber strength stimulation. Additionally, both during A- and C-fiber strength stimulation, the fluorescence intensity of neuronal cell bodies increased with stimulus frequency. Scale bar: 100 µm. See also Figure 3. 10.1523/ENEURO.0417-17.2018.video.1

When the stimulation frequency was sufficiently low and the image acquisition rate high, a fluorescence signal was seen in response to the first of a train of stimuli as well as subsequent arriving impulse in the majority of neurons, revealing sufficient sensitivity to detect single action potentials (Fig. 3*C*,*D*). To ensure that only action potentials generated through stimulation of the sciatic nerve were recorded, the stimulated nerve was transected distally to the stimulation site. This procedure ensured an absence of contaminating spontaneous peripheral inputs, confirmed by the lack of response to paw pinching. The magnitude of this unitary response varied greatly, in some cases exceeding 100% of baseline fluorescence, and typically had a rise time of around 200 ms and an exponential decay with a time constant of ∼700 ms.

As expected, A-fiber strength stimulation mostly activated larger DRG cell bodies while higher, C-fiber strength stimulation recruited new, smaller cells (Fig. 3*E*).

Out of a sample of 48 neurons detectably responding to A-fiber strength stimulation at 5 Hz (summated signal), 40 neurons also showed a detectable signal at 1 Hz. Interestingly, in a sample of 62 cells detectably responding to C-fiber strength stimulation at 5 Hz, all were also seen to respond at 1 Hz frequency. Thus, in the large majority of cases, we are able to detect the fluorescence change associated with a single action potential. However, it should be noted that individual action potentials were here detected by eye and statistical cutoff values to detect single action potentials are likely to be less precise. This is due to the relatively small change in fluorescence intensity generated by non-summating action potentials.

### Assessing polymodality in primary afferents

To assess the level of polymodality within a neuronal population, all thermally responsive neurons were assessed for their responses to the opposing thermal modality (e.g., neurons observed responding to heat were similarly assessed for their response to cold), as well as their mechanical sensitivity.

**Figure 4.**
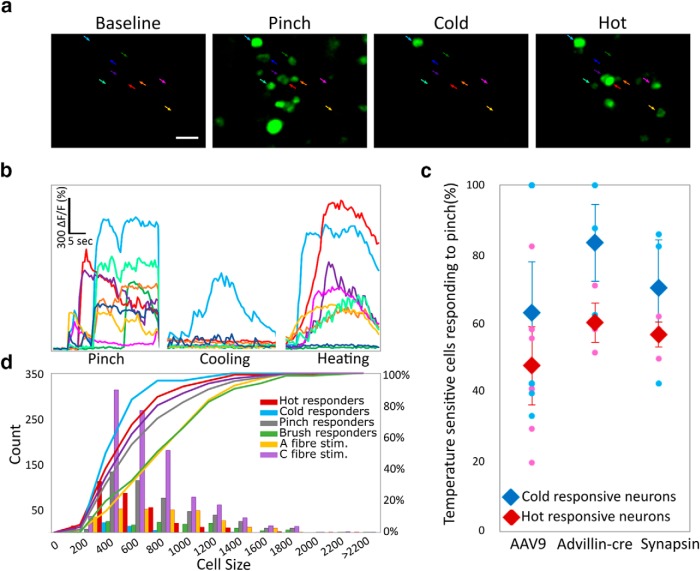
Polymodality is common in primary afferents. ***A***, Example images of DRG neurons responding to mechanical and thermal stimulation of the ipsilateral plantar paw. Scale bar: 50 µm. ***B***, Color coded example traces of neurons highlighted in ***A***. ***C***, Percentage of thermally sensitive DRG neurons responding also to noxious mechanical stimulation. Similar percentages of polymodal (thermally and mechanically sensitive neurons) were detected when using AAV9 to deliver GCaMP through intrathecal injections (*n* = 6) or when expressing GCaMP transgenically only in DRG neurons through GCaMP floxed mice expressing cre under the Advillin promoter (peripheral neurons; *n* = 3) or when expressing GCaMP in all neurons through the Snap25 promoter (*n* = 3). Total *n* = 1138 neurons. Blue data points show the percentage of cold sensitive neurons responding to pinch while red data points indicate the percentage of hot responding neurons also responding to pinch. Diamonds display the mean ± SEM, circles indicate single mice. ***D***, Cell size distribution of cell bodies that respond to changes in temperatures versus cell bodies that respond to A-fiber strength stimulation, C-fiber strength stimulation, brush and pinch, and the cumulative sum of their sizes. Cell bodies responsive to A-fiber strength stimulation, C-fiber strength stimulation, brush and pinch are significantly larger compared to somata which respond to changes in temperature: two-sample Kolmogorov–Smirnov test, total *n* = 1166 neurons in *n* = 4 mice, *p* < 0.001 for all comparisons against temperature sensitive cell bodies, except temperature-responsive neurons versus C-fiber stimulation where *p* = 0.019. See also Video 2 for a representative recording of polymodal responses in the DRG.

A small proportion of heat-responding neurons also responded to decreases in temperature (∼4% in 444 heat-responsive neurons, across six animals), while a larger proportion of cold responding neurons (∼40% in 31 cold-responsive neurons, across six animals) also encoded an increase in temperature (Fig. 4*A*,*B*). However, due to the very small number of cold-responsive neurons, these percentages varied greatly between experiments. Around 55% of these temperature-responsive neurons were also responsive to pinch (Fig. 4*A*–*C*), revealing a subset of multimodal-temperature-sensitive neurons. See also Video 2 for a representative recording of polymodal responses in the DRG.

Video 2. Polymodality in DRG neurons. L4 DRGs response to peripheral cooling, heating, and noxious pinching of the ipsilateral paw. Neurons responding to pinching of the ipsilateral paw are green, neurons responding to heating are red and neurons responding to cooling are blue. A high level of polymodality is observed. Scale bar: 50 µm. For quantification, see also Figure 4. 10.1523/ENEURO.0417-17.2018.video.2

Size distribution showed that thermosensitive DRG somata were significantly smaller than cell bodies of the same cohort of animals that responded to stimulation by brush and A-fiber strength electrical stimulation (Fig. 4*D*). Indeed, cold-sensitive neurons represented the smallest pool of cell bodies, while neurons that were pinch-sensitive or responding to C-fiber strength stimulation were of average size, in agreement with the non-discriminative nature of these stimuli.

To ensure that our labeling technique did not lead to a selection bias, the responsiveness of temperature sensitive neurons to mechanical stimulation (pinch) was also assessed in two different lines of GCaMP6 transgenic mice (Fig. 4*C*). In all three approaches used (i.e., intrathecal injections of AAV9 carrying the GCaMP6 transgene, vs two types of transgenic animals expressing GCaMP6 constitutively in all neurons or conditionally only in peripheral neurons), polymodality was maintained above 48% (Fig. 4*C*).

### Assessing the effect of an intraplantar injection of formalin on prolonged DRG activity

The formalin test is very widely used in pain research, but the mechanisms underpinning the responses are not completely understood. To address this question, we injected 20-µl formalin (1.85% in saline) and vehicle into the plantar surface of the hind paw, while simultaneously imaging the ipsilateral DRG.

**Figure 5.**
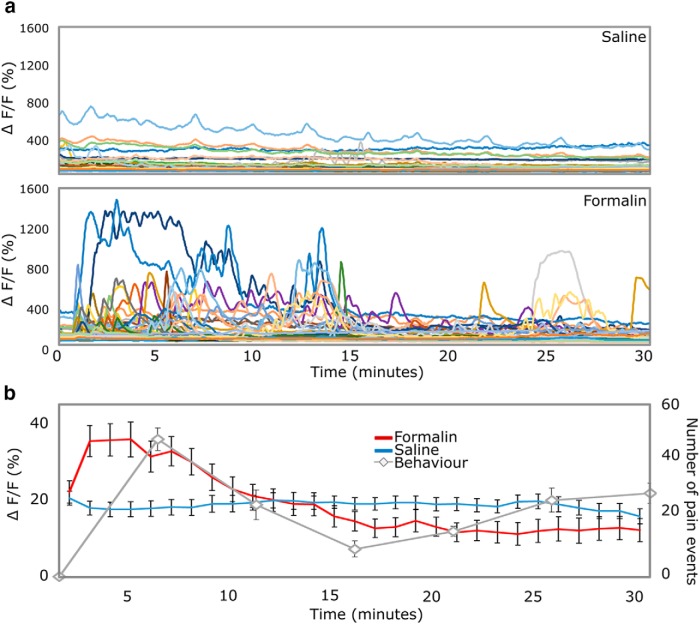
DRGs play a defining role in the generation of pain behavior in the formalin test. ***A***, Example traces of GCaMP fluorescence 0–30 min after intraplantar injection of saline (0.9%) and formalin (1.85%) in the same neurons. ***B***, Average response of DRG neurons (*n* = 666 neurons in *n* = 5 mice, all subjected to saline and formalin) and behavioral response in mice (*n* = 8) after injection of saline and formalin into the plantar surface of the hind paw. Cellular response was averaged over 1 min and across neurons. Pain behavior was assessed as the number of pain events displayed by the mouse in 5-min increments. All data displayed as mean ± SEM. Repeated-measures ANOVA, interaction of treatment (formalin vs saline) with time: *F*_(28,18,620)_ = 16.607, *p* < 0.001.

An injection of saline produced, as expected, a brief burst of activity, presumably from the pressure and/or damage associated with the injection. In contrast, the formalin injection induced a significantly greater response, much of it with a delayed activation (Fig. 5), with some neurons showing brief and sometimes repeated bursts of activity a few minutes after formalin injection (Fig. 5*A*). Indeed, the composite activity of DRG neurons mirrored pain behavior up to the second peak (Fig. 5). However, following the interphase, the average firing dropped considerably. Bouts of activity were still presented, mainly in small neurons, continuing through the remainder of the observation period. Some have suggested that formalin induces a “wave” of activation as it spreads spatially in the tissue. We did not observe a sequential activation of different cell bodies. Rather individual neurons could stop and start, apparently randomly throughout this latter phase. Because this activity was of a low level and asynchronous among different neurons, the average rate of firing at these later times was not statistically different from saline treated animals.

### UVB irradiation leads to an increased sensitivity of thermally responsive neurons

**Figure 6.**
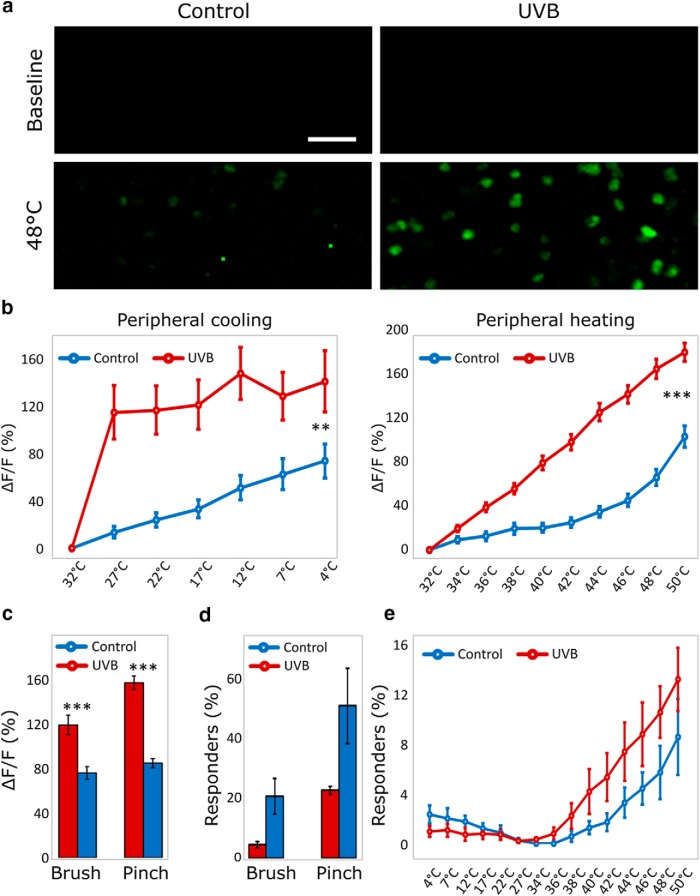
UVB irradiation increases the responsiveness of peripheral neurons. ***A***, Representative images of DRG neurons in mice stimulated with 32°C (baseline) and 48°C on the ipsilateral plantar surface, 48 h after UVB irradiation or control anesthesia. Scale bar: 100 µm. ***B***, The response intensity of neurons stimulated thermally 48 h after UVB irradiation or control anesthesia. The response of neurons to both warming (*n* = 393 neurons) and cooling (*n* = 71 neurons) of the ipsilateral paw is significantly greater in UVB-irradiated animals compared to controls. Differences were compared between groups in a split-plot ANOVA: For warming *F*_(1391)_ = 28.573, *p* < 0.001 and for cooling *F*_(1,69)_ = 11.421, *p* = 0.001. ***C***, Response intensities of neurons stimulated mechanically (brush: *n* = 222 neurons; pinch: *n* = 787 neurons) 48 h after UVB irradiation are significantly greater as compared to neurons in sham irradiated mice (independent sample *t* test, equal variances not assumed; for brush *t*_(149.742)_ = 4.112, *p* < 0.001. For pinch *t*_(730.186)_ = 9.848, *p* < 0.001). ***D***, The percentage of neurons responding to mechanical stimulation was not significantly different in UVB-irradiated animals compared to controls (independent sample *t* test, equal variances not assumed; *t*_(2.112)_ = 2.696, *p* = 0.108 for neurons responding to brush and *t*_(2.0456)_ = 2.216, *p* = 0.154 for neurons responding to pinch). ***E***, The percentage of neurons responding to both warming and cooling of the ipsilateral paw was not significantly different in UVB-irradiated animals compared to controls (between groups difference in a split-plot ANOVA, *F*_(1,5)_ = 1.6, *p* = 0.262). ***p < 0.001, **p < 0.002. For all experiments data displayed as mean ± SEM, *n* = 7 mice (three control, four UVB).

UVB irradiation is used as a model of inflammatory pain in humans and animals. We assessed the consequences of this treatment on the responsiveness of sensory neurons. We found that UVB inflammation consistently led to a striking increase in the responsiveness of temperature sensitive neurons (Fig. 6*A*,*B*). Indeed, warming of the injured paw led to significant increases in the responses of affected primary afferents which now revealed a linear increase in response with increases in temperature. This is compared with much lower levels of activation after heating of the uninjured paw. Here, low levels of heating had very limited effects on neuronal activity and much higher levels of stimulation were needed to achieve significant increases in neuronal responses (Fig. 6*A*,*B*). The sensitization of the peripheral nervous system was not limited to heating but also included enhanced sensitivity toward cooling of the injured paw. Even small decreases in temperature (e.g., to 27°C) resulted in much greater activation of responding neurons during sterile inflammation as compared with responses in healthy control animals (Fig. 6*B*). In addition, responses to mechanical stimuli, both noxious (pinch) and innocuous (brush), were significantly enhanced after UVB irradiation (Fig. 6*C*).

Interestingly, UVB irradiation did not lead to a significant increase in the percentage of neurons responding to thermal or mechanical stimulation of the irradiated paw, when compared to control anesthetized mice (Fig. 6*D*,*E*).

## Discussion

Here, we use a new technique for the visualization and study of hundreds of peripheral neurons simultaneously *in vivo*. Using advanced genetically encoded calcium indicators, together with standard confocal microscopy, we were able to assess population responses of DRG neurons to electrical, thermal, mechanical and chemical stimuli, providing new insights into the processing of sensation and nociception in the peripheral nervous system during health and injury. While there have been a few reports using similar approaches, this is the first to report on large neuronal populations (typically >200 cell bodies in a single preparation), acquired at a subsecond frame rate.

The exposure and stabilization of the DRG involved a laterally extended laminectomy. Due to the encasing bony structure of the vertebrae, sufficient stabilization only required two vertebral clamps on either side of the exposure. However, the orientation of the DRG around the spinal cord meant that aligning the entire structure into one focal plane was challenging. As a result, confocal microscopy with an open pinhole was considered beneficial over two-photon microscopy in this instance. This approach is complementary to other methods, using both multi- and single-photon microscopes, aimed at increasing the acquisition of information in space while maintaining temporal resolution. Such methods include for example the use of a piezoelectric device (Callamaras and Parker, 1999), Bessel beams (Thériault et al., 2014), acousto-optical deflectors (Reddy and Saggau, 2005), multi-spot excitation (Bewersdorf et al., 1998; Kurtz et al., 2006), as well as various adaptations of scanning patterns (Göbel et al., 2007; Lillis et al., 2008). While our approach loses some of the added benefits often associated with two-photon microscopy, including greater penetration depth and reduced photo damage, it requires no modification of the microscope or software and provides a large dynamic range over which to detect changes in calcium signal, with a favorable signal-to-noise ratio and reduced effects of movement artefacts. In addition, the signal remained stable across long imaging sessions (>2 h) with no detected bleaching.

The functionality of our system was validated by electrical stimulation of the sciatic nerve, activating DRG neurons in a predictable and reproducible manner (Fig. 3). The results of our experiments indicate that GCaMP6 fluorescence in DRG neurons is a sensitive reflection of neuronal activity, with good temporal resolution. Interestingly, our approach resulted in much greater numbers of neurons detected at much faster rates, compared to previously published works (Emery et al., 2016; Kim et al., 2016; Smith-Edwards et al., 2016). As expected, the majority of L4 DRG neurons responded to electrical stimulation in a frequency-dependent manner: low-threshold electrical stimuli recruited fewer neurons in the DRG, and these tended to have larger DRG somata. In contrast, high-threshold stimulation activated both the same large neurons and additionally many more (typically small diameter) neurons. Moreover, the GCaMP6 reporter was sensitive enough to detect calcium transients in the DRG elicited by single action potentials generated in the periphery.

At stimulation frequencies above 1 Hz, temporal summation of the calcium transient meant that the fluorescence intensity also served as a proxy for the rate of action potential firing. The magnitude of calcium transients for a given level of activity varied between neurons. Some of this presumably represents variation in expression levels of GCaMP6s in different cell bodies, but it may also represent differences in calcium handling by different cell types (Scroggs and Fox, 1992; Malmberg and Yaksh, 1994; Westenbroek et al., 1998; Simons et al., 2009; Gemes et al., 2010).

Having established the sensitivity and specificity of the technique, we next investigated several aspects of the neurobiology underpinning sensory perception. Due to the novelty of the method, and several technical challenges it poses, it is essential to carefully examine novel claims that contrast traditionally held views. Indeed, one such claim suggests that polymodality, traditionally seen using microneurography, electrophysiology, multiple lines of *in vitro* evidence, and now also *in vivo* GCaMP imaging, was due to inflammation introduced through the use of invasive techniques (Emery et al., 2016). However, our data show that this is unlikely to be an adequate explanation as the use of three different labeling techniques (including transgenic expression of GCaMP similar to that used by Emery et al., 2016) all resulted in similar levels of polymodality. It is possible that since Emery and colleagues imaged a smaller number of cell bodies, their results might have been skewed in unexpected ways, missing one of the most essential benefits of peripheral imaging, which is the collection of large sets of unbiased observations. A further reason for the discrepancy between their and our findings could be related to the statistical definitions of polymodality. The application of mechanically noxious stimuli also includes the application of non-noxious touch, necessarily activating a number of non-nociceptive neurons. By calculating the percentage of polymodal neurons using mechanical stimulation as the denominatory (i.e., percentage of thermally responsive neurons per mechanically responsive cell), Emery et al., are very likely to have significantly underestimated the percentage of polymodal neurons. In this manuscript, mechanical sensitivity is the numerator, meaning we calculated the percentage of mechanically responsive neurons per thermally responsive cell. This avoids diluting our results with touch sensitive neurons. We are perhaps still unavoidably underestimating the percentage of polymodal neurons by stimulating slightly different receptive fields with different stimulation modalities. We can therefore only comment on the minimum number of polymodal neurons, which is still significantly greater than that reported by Emery et al. (2016).

The visualization of large numbers of cell bodies with good temporal resolution can provide a reliable system with which to study the involvement of the peripheral nervous system in several acute and chronic pain conditions. The formalin test is one of the most widely used models for the study of pain, particularly in the pharmaceutical industry. Controversy still exists as to its mechanism of action, particularly relating to the relative role of the peripheral versus CNS. It has variably been suggested that the second phase is generated directly by afferent activity (McCall et al., 1996) or by sensitization of the spinal dorsal horns (Coderre et al., 1990, 1993; Malmberg and Yaksh, 1992), or even supraspinal structures (Vaccarino and Melzack, 1989). The ability to visualize hundreds of DRG neurons simultaneously was well suited to investigate the relative role of the peripheral network in the generation of the formalin response.

Our results show high levels of DRG activation soon after an injection of formalin with a peak activity 5 min postinjection (Fig. 5). The cellular response was enhanced and delayed relative to saline, in line with previous work (Puig and Sorkin, 1996). We also found that neurons responded very heterogeneously. A small number fired tonically for long periods of time postinjection, and were likely responding to distention and/or damage associated with the injection (since similar responses were seen after saline injections). Beyond these, there were many neurons that were activated selectively by formalin. Some were activated soon after the injection, while others showed a more delayed response, either in the form of a single “burst” or of multiple cycles of activity across the observed period. In contrast to previous electrophysiological data (McCall et al., 1996; Henry et al., 1999), our recorded neurons did not seem, in aggregate, to constitute two clear phases of activity. Rather we saw strong activation of many neurons corresponding to the first phase of behavior, followed by low levels of intermittent firing seen in small numbers of predominantly very small neurons. We interpret this as evidence that the initial activation of neurons following a formalin injection might be the sensory drive for the first phase response. This activity also sensitizes dorsal horn neurons so that low levels of asynchronous activity in the periphery is able to generate a second phase in pain behavior, which is thought to be sustained by central sensitization and responsive to the application of pharmacological blockers in the spinal cord (for review, see McMahon et al., 1993). The low level of peripheral activity seen after a saline injection will impinge on a non-sensitized spinal cord, and hence does not initiate a long lasting behavioral change. In support of this view, early electrophysiological studies reported only low levels of C-fiber activity during the second phase of the formalin test (McCall et al., 1996). Such low levels are likely to be difficult to see clearly with calcium imaging but may provide sufficient input to activate a sensitized central pain pathway.

One interesting and highly translatable model of persistent pain is UVB-induced sterile inflammation. UVB irradiation leads to consistent swelling and hyperalgesia 48 h after injury. Here we were able to directly assess the involvement of the peripheral nervous system in the resulting hyperalgesia (Fig. 6). Indeed, we were able to visualize significant increases in sensitivity to peripheral heating and peripheral mechanical stimulation (both noxious and innocuous) as reported previously using electrophysiological methods (Bishop et al., 2010). Additionally, we also demonstrate for the first time, to the best of our knowledge, the involvement of the peripheral nervous system in UVB induced cold sensitivity. Indeed, we show that even small decreases in temperature had much more profound effects on peripheral responses after UVB irradiation as compared with responses from control animals. Interestingly, no significant differences were observed in the percentage of neurons responding to mechanical or thermal stimuli. This suggests limited unmasking of silent neurons with this model of sterile inflammation, and instead implicates mainly an increased activation of responding neurons.

It should be noted that *in vivo* imaging provides a significant advantage over traditional electrophysiological techniques in questions of cold processing. The limited number of cold sensitive primary afferents provides a practical barrier for the assessment of changes in peripheral cold processing using stochastic single cell approaches. Instead, the ability to sample hundreds of neurons simultaneously, as shown here, provides an excellent opportunity to investigate populations of rare neurons, including, but not limited to, cold-sensitive afferents.

In conclusion, we significantly enhanced the spatial and temporal sensitivity of a novel technique applied to the large-scale study of the peripheral nervous system. Using this technique, we were able to confirm the traditionally held, but recently challenged, view of polymodality in the peripheral nervous system. Additionally, we revealed new roles for peripheral processing in acute and persistent pain states.

## Synthesis

Reviewing Editor: Karen Davis, Krembil Research Institute and University of Toronto Decisions are customarily a result of the Reviewing Editor and the peer reviewers coming together and discussing their recommendations until a consensus is reached. When revisions are invited, a fact-based synthesis statement explaining their decision and outlining what is needed to prepare a revision will be listed below.

This study could advance the field by it's report of an improved imaging technique. It also confirms the long standing view in the field that many primary afferent neurons are indeed Polymodal, a view that was recently challenged by a report in a high-profile journal by Emery, John Wood and colleagues, who happen to be investigators/colleagues at the same Institute as the current authors. While the ability to phenotype large numbers of sensory neurons in the same animal has been presented in this previous publication, this is an important area of sensory neuroecience which would greatly benefit from the present analysis. That they have been able to elucidate a phenotype much more compatible with a long and extensive literature on somatosensory physiology supports this conclusion.

The present work presents both a broadly useful technique for the study of populations of somatosensory neurons that is likely to be used by other investigators as well as providing compelling data on the response properties of somatosensory neurons, at the population level. I have but minor suggestion. Previous reviews have questioned why these authors do not see late phase responses to formalin. Work by Levine's group (single fiber studies) that of Sorkin and colleagues demonstrated activity in C-fibers in phase 2, the level of activity was extremely small relative to that in phase 1. A note to this effect might clarify this issue.

## Author Response

REVIEWS

Reviewer #1 (Synthesis Statement for Author (Required)):

This study could advance the field by it's report of an improved imaging technique. It also confirms the long standing view in the field that many primary afferent neurons are indeed Polymodal, a view that was recently challenged by a report in a high-profile journal by Emery, John Wood and colleagues, who happen to be investigators/colleagues at the same Institute as the current authors. While the ability to phenotype large numbers of sensory neurons in the same animal has been presented in this previous publication, this is an important area of sensory neuroecience which would greatly benefit from the present analysis. That they have been able to elucidate a phenotype much more compatible with a long and extensive literature on somatosensory physiology supports this conclusion.

The present work presents both a broadly useful technique for the study of populations of somatosensory neurons that is likely to be used by other investigators as well as providing compelling data on the response properties of somatosensory neurons, at the population level. I have but minor suggestion. Previous reviews have questioned why these authors do not see late phase responses to formalin. Work by Levine's group (single fiber studies) that of Sorkin and colleagues demonstrated activity in C-fibers in phase 2, the level of activity was extremely small relative to that in phase 1. A note to this effect might clarify this issue.

We are very grateful for the reviewer's comments. We have adjusted the manuscript to include reference to previous works with low levels of C-fibre activity in phase 2 of the formalin response (see highlighted text in manuscript).
